# Advances in the Study of Probiotics for Immunomodulation and Intervention in Food Allergy

**DOI:** 10.3390/molecules28031242

**Published:** 2023-01-27

**Authors:** Yan-Yan Huang, Yan-Tong Liang, Jia-Min Wu, Wei-Tong Wu, Xin-Tong Liu, Ting-Ting Ye, Xiao-Rong Chen, Xin-An Zeng, Muhammad Faisal Manzoor, Lang-Hong Wang

**Affiliations:** 1 College of Food Science and Engineering, Foshan University, Foshan 528225, China; 2 Guangdong Provincial Key Laboratory of Intelligent Food Manufacturing, Foshan University, Foshan 528225, China

**Keywords:** probiotics, intestinal flora, food allergy, immunity

## Abstract

Food allergies are a serious food safety and public health issue. Soybean, dairy, aquatic, poultry, and nut products are common allergens inducing allergic reactions and adverse symptoms such as atopic dermatitis, allergic eczema, allergic asthma, and allergic rhinitis. Probiotics are assumed as an essential ingredient in maintaining intestinal microorganisms’ composition. They have unique physiological roles and therapeutic effects in maintaining the mucosal barrier, immune function, and gastrointestinal tract, inhibiting the invasion of pathogenic bacteria, and preventing diarrhea and food allergies. Multiple pieces of evidence reveal a significant disruptive effect of probiotics on food allergy pathology and progression mechanisms. Thus, this review describes the allergenic proteins as an entry point and briefly describes the application of probiotics in allergenic foods. Then, the role of probiotics in preventing and curing allergic diseases by regulating human immunity through intestinal flora and intestinal barrier, modulating host immune active cells, and improving host amino acid metabolism are described in detail. The anti-allergic role of probiotics in the function and metabolism of the gastrointestinal tract has been comprehensively explored to furnish insights for relieving food allergy symptoms and preventing food allergy.

## 1. Introduction

**Figure 1:**
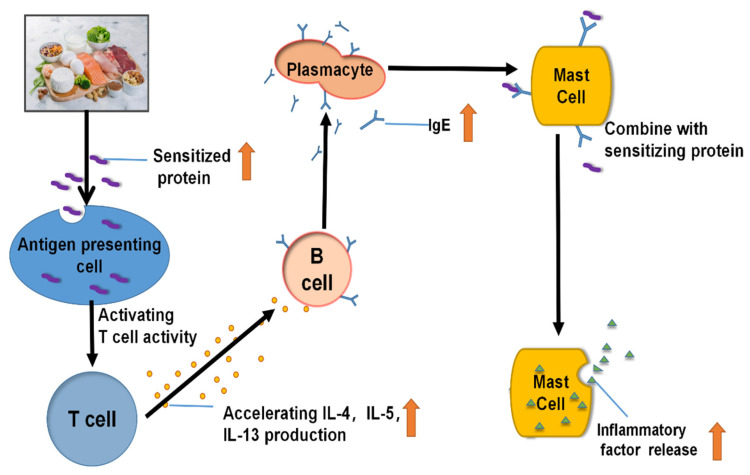
Immune mechanism of food allergy.

In food allergy, bio-molecules such as food proteins act as antigens to stimulate the abnormal immune response, usually accompanied by vomiting, diarrhea, blood in the stool, eczema, or weird reactions in the gastrointestinal tract. Their severity can lead to shock or even death [1]. The proposed immune mechanism of food allergy is shown in Figure 1. However, combating food allergy is turtlenecked owing to the complexity and diversity of food allergens. We can see that avoiding food allergens is normally considered the most effective control measure. Research data show that approximately 1 billion people worldwide suffer from allergies. The prevalence of allergic diseases has increased substantially in recent years, with 30–40% of the global population suffering from one or more allergic diseases. In China, for example, the prevalence of food allergies in the Chinese population has risen by 60% over 10 years. A survey showed that 22% of allergic patients are allergic to milk, and infants aged 0–3 are more likely to be allergic to milk than to various food allergens [2].

Additionally, 27% of patients are allergic to eggs. Infants are more susceptible to eggs than children. Interestingly, contrary to egg and milk allergies, fruit allergies are more common in children than in infants [3]. Probiotics are a group of active beneficial microorganisms that can colonize the host gut to improve the host’s intestinal microorganisms balance and exert beneficial effects [4]. Probiotics can not only maintain the dynamic balance of intestinal flora but also enhance the immune function of intestinal mucus, so it is expected that probiotics can be applied to the clinical treatment of allergic diseases [5]. Probiotics have also been shown to alleviate the allergic reactions caused by such food allergens. Probiotics significantly affect the ability to improve the human body’s immune response by maintaining the stability and balance of intestinal flora, strengthening the intestinal barrier, and enhancing the body’s metabolism, to improve immunity to suppress the allergy [6]. Food allergy has now been identified as a thorny issue that seriously endangers public health and poses a considerable food safety risk. Thus, an in-depth study of food allergy is necessary for worldwide public health safety. This review summarized typical food allergens’ characteristics and introduced probiotics’ application in various allergic foods. In addition, the relationship between probiotics and the immune system is also discussed, which means that probiotics can treat and prevent food allergies through immunomodulation. Based on probiotics’ limitations, postbiotics can also potentially interfere with food allergies. The study proposed a new idea for alleviating food allergy symptoms and their allergies.

## 2. Allergy Mechanism of Various Food Allergens

More than 9% of food allergens belong to soybean, dairy, aquatic, eggs, and nuts product proteins, as shown in Table 1 [2]. Food allergy is mainly mediated by Immunoglobulin E (IgE) and Immunoglobulin G (IgG), which leads to an immune response. Here are some common allergy-causing foods which will introduce the mechanism of allergy.

### 2.1. Soy Products

Plant-based food allergens are dominated by soybean products, such as tofu, and its related products formed by solidifying soybean milk. Bean is rich in nutrients. Bean protein is an important source of plant protein and is widely used in the food industry. Soy proteins are quite similar to animal proteins in amino acid composition, and their amino acid proportion in the organism are remarkably similar, so they are more efficiently utilized for digestion and absorption [15]. Soybean is considered a vital allergenic food due to the variety of antigenic proteins, such as glycinin, β-conglycinin, Gly m Bd 60 K, Gly m Bd 30 K, and Gly mBd 28 K [16]. These allergenic proteins are divided into two superfamilies: prolamin and cupin. Gly m 5 (7S globulin) and Gly m 6 (11S globulin) are two cupin superfamily allergens found in soybean. The cupin protein superfamily is thermally stable and can form an immunogenicity-boosting aggregate [17]. The allergenic 2S albumin nGly m 8, a protein significantly connected with allergic responses and an essential indication of allergic reactions to beans, is a member of the Prolamin superfamily. This is because 2S albumin has a well-defined three-dimensional structure and responds consistently to heat processing and protease catabolism [18]. Hao et al. specified that protein subunit and multiple IgE and IgG binding sites demonstrate that the primary allergic mechanism of soy products is mainly IgE-mediated type I hypersensitivity reaction to mast cell activation and IgG-mediated type II hypersensitivity reaction [19].

A study was conducted to construct an inflammatory model of Caco-2 cells induced by LPS (lipopolysaccharide), intervening with different *Lactobacillus Beiderbecke* subsp. *Bulgaricus* and investigating the effects of other LPS on inflammation in intestinal epithelial cells by evaluating the levels of IL-6 and IL-8 secretion in cells. The results showed that both live and inactivated bacterial bodies could inhibit the elevated levels of IL-6 and IL-8 induced by LPS, thereby alleviating the inflammatory response induced by LPS [20]. A study has demonstrated that soy milk fermentation with *Bacillus subtilis*, *Rhizopus oryzae*, *Saccharomyces cerevisiae*, and *Lactobacillus helveticus* can reduce allergies to soy protein to varying degrees. It is worth mentioning that soymilk fermentation with *Lactobacillus helveticus* substantially reduced allergies and effectively removed the off-flavors and bitterness of soy milk after fermentation [21]. Therefore, the fermentation of soybeans using LAB (lactic acid bacteria) will be the focus of future research on reducing food allergy immunity.

### 2.2. Dairy Products

Bovine milk protein is an essential nutrient for the nourishment of infants and children. It has been identified by the World Health Organization (WHO) as one of the eight primary allergenic foods. Dairy allergen proteins are dominated by casein, β-lactoglobulin, and α-lactalbumin, and casein is mainly composed of four proteins, αS1-casein, αS2-casein, β-casein, and κ-casein [8]. The higher the proportion of casein in the total protein, the more potentially allergenic. This is because casein allergic reactions may trigger a Th2 response, disrupting the Th1/Th2 immune balance and predisposing the infant to sensitization by other milk proteins such as β-lactoglobulin and α-lactoglobulin [22]. These proteins have a complex tertiary structure and similarities in peptide chain conformation, recognizing similar T and B cell epitopes [23]. Dairy products are closely associated with food allergy and dysregulation of the body’s immune response, including IgE-mediated activation of mast cells and eosinophils, abnormal differentiation of CD4 lymphocyte subsets, and excessive production of pro-inflammatory cytokines [24]. Milk allergy is generally believed to be mainly an IgE-mediated allergic reaction [25].

Hydrolyzed protein has been shown in studies to lessen the development of allergy reactions in dairy products by causing allergen protein to lose its IgE binding epitope [26]. Previously, it was observed that when *Lactobacillus delbrueckii* subsp. *bulgaricus* CRL 656 was utilized for fermentation, the whey allergen in skim milk and whey extract was somewhat reduced [27]. Furthermore, hydrolyzed protein has been shown to lower permeability in an external model of the intestinal tract, which aids in barrier function and antigen absorption. Milk protein hydrolysate, in particular, appears to have anti-inflammatory properties. In the in vitro assay to measure the potential immunomodulation, the hydrolysate of *Lactobacillus delbrueckii* ssp. *lactis* 92202, *Lactobacillus helveticus* 9201, and *Lactobacillus delbrueckii* ssp. *bulgaricus* 92059 produced anti-inflammatory activity in the presence of TNF-α [28]. Due to the increasing attention to intestinal probiotic therapy, treating food allergies by intestinal flora is expected to be a new approach to improving the problem of dairy allergy.

### 2.3. Aquatic Product

Aquatic products are processed and manufactured from animals and plants native to seawater and freshwater. Aquatic products are highly nutritious and rich in minerals, calcium, phosphorus, potassium, and vitamins; in contrast, they are potent in causing food safety risks. Proteins, such as parvalbumin, ichthulin, tropomyosin, aldolase A, and β-enolase, are the primary allergens of aquatic products [10]. Parvalbumin consists of six α-helices (A–F) and short segments of antiparallel β-sheets, which are calcium-binding proteins. They can be divided into two phylogenetic spectra, α and β, based on differences in isoelectric points. β-small albumin contains more acidic amino acid residues and has an isoelectric point (pI) below 4.8 [29]. Tropomyosin is the major allergen of aquatic crustacean products, and as a highly stable α-helical coiled-coil homo-dimeric protein, it retains its allergenicity in high-temperature and high-pressure processing [30]. The main allergenic fragments of these proteins are water-soluble glycoproteins with good resistance to heat, acid, and protease digestion. Immunostimulatory factors in the food may also contribute to this allergenic effect [31]. Food allergy is characterized by immune cross-reactivity, and the antigen epitope of the allergen is the basis of causing an allergic reaction. Epitope composition and spatial conformation characteristics determine its antigen specificity [32].

Probiotics can activate the phagocytosis of macrophages and regulate the immune balance between Th1-Th2 to enhance the collective nonspecific and specific responses to improve disease resistance [33]. A study constructed an animal model of the South American white shrimp muscle protein allergy in Balb/c rats and compared the therapeutic effect of *Bifidobacterium longum* and *Bacillus coagulans*. The results demonstrated that *Bifidobacterium longum* and *Bacillus coagulans* inhibit mast cell degranulation and reduce specific IgE and IgG1 antibody content, which indicated that LAB could both promote antibody conversion and inhibit Th2 immune response [34].

### 2.4. Poultry and Egg Products

Eggs are an integral part of daily life and are highly prized for their affordability, nutrition, and protein content. Egg allergy IgE-mediated can involve the skin, gastrointestinal, and respiratory tract, despite causing relatively some acute allergic reactions [12]. Most children are allergic to egg white, which contains four major allergens: ovomucoid, ovalbumin, ovotransferrin, and lysozyme [13]. Ovomucoid is stable from heat to acid, and the resulting sIgE will cause a severe immune response, with the highest sensitization among the four allergens [35]. In contrast, the most abundant ovalbumin showed poor thermal stability [36]. The allergenicity of poultry eggs has always been a hot research topic in the scientific community. Using a healthy population as a control group, subtle differences were found in the gut microbiota composition of egg-allergic patients. Specifically, *Lactococcus* and *Ruminococcus* were enriched in egg-allergic subjects, while *Leuconostoc* was enriched in healthy children [37]. Therefore, the proportion of *Lactobacillus* in the composition of microorganisms will also change during the development of food allergy.

Recently, safe and effective allergen-specific subcutaneous immunotherapy has been developed to combat food allergies using recombinant hypoallergenic proteins instead of food extracts [38]. A study amplified by polymerase chain reaction with a C-terminal hemagglutinin epitope-tagged the gene encoding chicken serum albumin, which was successfully expressed in the yeast *Kluyveromyces lactis* protein expression system [39]. Experiments show that recombinant proteins expressed in probiotic bacteria have similar strong immunomodulatory properties of allergen proteins, which can stimulate immune cell proliferation in allergic patients [40]. This implies that probiotics have the potential to act as allergen vaccine carriers.

### 2.5. Nut Products

Nuts are highly nutritious foods that quickly cause allergies [14]. Allergic reactions to nuts can be divided into class I food allergy and class II food allergy, which IgE mediates. Of note is that class II food allergy is associated with sensitization to pollen allergens [41]. In the case of hazelnut, for example, there is a similarity in protein sequence between the Bet v 1 allergen in birch pollen and the Cor a 1 allergen in hazelnut, which would increase the odds of cross-reactivity [42]. Cross-reactivity is possible with consuming multiple nut allergens, such as walnuts, pecans, cashews, and pistachios [43]. However, the extent of cross-reactivity between different varieties of nuts is still unknown at this stage and needs to be studied. Most of the proteins involved in tree nut allergy belong to the family of proteins such as 2S albumin, vicilin, legumin, and non-specific lipid transfer proteins (nsLTPs). 2S albumin and nsLTPs contain large amounts of sulfur-rich amino acids that form disulfide bonds that promote structural stability and make them resistant to heat treatment and enzymatic processing [44].

To strictly avoid the possibility of being allergic to other nuts, in the past, once a certain nut allergy was diagnosed, patients were forbidden to eat all nuts. Therefore, homologous analysis of nut allergens can accurately intervene in allergy [14]. Probiotics inhibit the proliferation of pathogenic microorganisms in their ability to metabolize polyunsaturated fatty acids (PUFA) in the intestine. PUFA can interfere with the production of dendritic cells, T cells, and IgE by B cells to resist the action of inflammatory factors caused by food allergy [45]. Liu et al., in exploring the fermentation process of walnut milk, found that *Lactobacillus plantarum* LP56 fermented walnut milk produced stable PUFA [46]. Reducing nut allergenicity through LAB fermentation will be a new idea to improve the nut food industry in the future.

## 3. Probiotics Regulate Immunity through Intestinal Flora

The intestine is a common site for nutrient absorption, colonization by commensal bacteria and localization of immune cells, and this spatial proximity facilitates their interactions to some extent. Upon comprehensive study of food allergy triggers, we further investigated the mechanism by which probiotics regulate the body’s immunity and thus food allergy through intestinal flora, and the effects of major probiotics on host food allergy are shown in Table 2.

### 3.1. Relationship between Probiotics and Intestinal Flora

The intestine is the body’s largest endocrine organ, and intestinal flora’s presence can ferment food to produce metabolites such as short-chain fatty acids, secondary bile acids, and amino acid metabolites [47]. These metabolites directly or indirectly affect the host gut and physiological health, and disorders of the intestinal flora may induce or aggravate food allergic reactions. A past study noted that the serum IgE level of mice and sterile mice with low diversity microbiomes would increase in early life, leading to the Th1/Th2 balance shifting to Th2, causing an allergic reaction [56]. Consequently, increasing the abundance or quantity of intestinal flora can effectively avoid food allergies. Probiotics in intestinal flora are the critical components that can regulate the composition of host intestinal flora in two ways, thus activating the endogenous microbial activity of the host and feeding back the body’s immune response. The first way is to directly change the structure of the inherent flora in the human intestinal tract by artificially regulating the intake of some probiotic products; second, it affects the metabolic activities of some intestinal flora and then changes the total content of short-chain fatty acids and bile acids in the intestinal tract [57]. Some studies have proved that probiotics can reduce the infection of intestinal pathogenic microorganisms by competing for nutrition or producing bactericidal factors to form colonization resistance, therefore reducing or preventing food allergy symptoms [58].

### 3.2. Probiotics Regulate the Immune Mechanism of Food Allergy through Intestinal Flora

Some probiotics in the human small intestine can produce organic acids, regulate the pH environment, and inhibit harmful bacteria growth. Moreover, they can produce antagonistic effects with harmful bacteria to change the structure of the intestinal flora. It can also regulate the body’s balance between Th1/Th2 to protect the normal immune function of human small intestinal skin mucosa [50,59]. Mouse experiments confirmed that *Lactobacillus paracasei* L9 had a regulatory effect on the Th1/Th2 imbalance of lymphocytes in mice with milk β-lactoglobulin allergy, which might be related to its efficacy in promoting the secretion of regulatory cytokines by DCs and increasing the number of CD4+FOXOP3+Treg cells [52,53,60].

*Lactobacillus* activates Th2 lymphocytes to produce large amounts of IL-5 when inducing a mucosal immune response. Influential IgA stimulating factors activate B lymphocytes to secrete IgA, which binds covalently to protein receptors produced by intestinal mucosal epithelial cells to produce the antibody secretory IgA (sIgA) [48]. The antibody sIgA is a secreted globulin that binds to pathogenic antigens and prevents their adsorption to the mucosal surface of the intestine [61]. In contrast to the study above, results of a study showed that *Lacticaseibacillus paracasei* L9 activated the TLR2/MAPK signaling pathway, promoted the differentiation of DCs to CD103+DCs and CX3CR1+DCs, enhanced the secretion of regulatory cytokines, increased the number of Foxp3+Treg and IL-10+Treg cells, corrected the Th1/Th2 imbalance in the allergic organism, and alleviated the Th1/Th2. The effect of the treatment was to alleviate the symptoms of cow’s milk protein allergy in mice [62].

### 3.3. Probiotics Regulate Immunity by Modulating the Intestinal Barrier

The small intestinal epithelium is the main part that constitutes the mucosal layer, and the intestine depends mainly on the differentiation of epithelial cells to reach the intestinal barrier [63]. Researchers have demonstrated that probiotics can improve the epithelial cytoskeletal structure to maintain bacterial cell wall permeability through information transmission [64]. It has been reported that oral administration of *Lacticaseibacillus rhamnosus* GG (LGG) promoted the rate of weight gain in newborn mice [65]. Studies of their intestinal microbiota revealed an accelerated rate of intestinal epithelial cell proliferation and differentiation compared to before weaning. Further research found that LGG, an early-life probiotic, could promote small intestinal growth and development to maintain intestinal stability by activating EGFR and other information pathways in intestinal epithelial cells after colonization [66].

As research progresses, it is increasingly evident that probiotic components could enhance intestinal epithelial barrier function [67]. Probiotics regulate intestinal epithelial homeostasis by promoting intestinal epithelial cell survival, enhancing barrier function, and stimulating protective responses while at the same time regulating host cell signaling pathways, including protein kinase B (Akt) signaling pathway, mitogen-activated protein kinase, and nuclear factor-κB [68]. Similarly, Rajput et al. found that probiotics stimulate epithelial cells and activate dendritic cells through toll-like receptors, producing cytokines [69]. Tests for intestinal barrier genes showed that *Bifidobacterium bifidum* could upregulate the expression of aromatic hydrocarbon receptor AhR and its downstream target gene Cyp1A1. In comparison, down-regulating inflammatory factors TNF-α, IL-6, and IL-1β and upregulating tight junction proteins ZO-1, Claudin-4, Occludin, and mucin Muc2 in colonic tissues [70].

### 3.4. Probiotics Regulate Host Immune Active Cells

The in-depth mechanisms study of food allergy revealed that gastrointestinal disorders increase the chance of food allergy occurrence [71]. Inflammatory bowel disease (IBD) and food allergy have similar clinical symptoms; clinical investigation of gut microbial changes with food allergies and IBD revealed similar changes in gut microflora abundance [72]. The relative abundance of microorganisms belonging to *Enterobacteriaceae*, *Veillonellaceae*, and *Streptococcus* increased. At the same time, there was a significant decrease in the relative abundance of microorganisms belonging to *Bifidobacteriaceae* and *Ruminococcaceae* [73]. Since intestinal inflammation and food allergy are characteristic of promoting each other simultaneously, intestinal inflammation symptoms can be alleviated by modulating immunoreactive cells to reduce the level of inflammatory factors in the treatment or prevention of food allergy [74]. Lymphocytes in allergic reactions can promote eosinophilia activation and release many inflammatory mediators to strengthen the allergic response [75]. Similar findings were reported by Srikham et al., who screened *Streptococcus salivarius* CP163 and *Streptococcus salivarius* CP208 in healthy breast milk. Both probiotic strains significantly induced immune responses through B and T lymphocyte activation [76]. Mast cells are also critical triggers in food allergy and inflammatory responses. Food allergens stimulate mast cell degranulation by releasing substances such as histamine and leukotrienes [50,77]. Earlier, researchers used ovalbumin to sensitize rats to food. After treatment with *Bifidobacterium bifidum*, the degree of intestinal mucosal destruction and mast cell degranulation was significantly alleviated [78]. These results demonstrate that probiotics can modulate the immunity and tolerance of host dendritic cells, epithelial cells, regulatory T cells, natural killer T cells, B cells, and immunoreactive cells.

### 3.5. Probiotics Improve Host Amino Acid Metabolism to Influence Immunity

Amino acids are an essential nutrient and are often used as the biochemical reaction substrate of immune cells to provide energy for immune cells. Probiotics can participate in amino acid metabolism, and the produced bioactive substances can change the host’s amino acid metabolism ability. Leucine (Leu) and Arginine (Arg) not only significantly enhance the expression of the major histocompatibility complex (MHC) on the surface of dendritic cells but also increase the relative gene expression of Interleukin-6 (IL-6) and Interleukin-12 (IL-12), which cause food allergic reactions [79]. However, Leu metabolism can be obstructed by oral administration of *Lactobacillus paracasei* PC-01. *Lactobacillus paracasei* PC-01 inhibits the activation of the mammalian target of rapamycin (mTOR) signaling pathway by amino acids in mammalian intestinal epithelial cells, alleviates the pro-food allergic response to amino acids, and consequently provides relief from a food allergy [80]. The tryptophan catabolism products in mammalian cells are kynurenine, tryptamine, and indole. There are three main pathways of tryptophan metabolism in the mammalian gut: the immune and epithelial cell tryptophan. The metabolism pathway is the kynurenine pathway dominated by indoleamine 2,3-dioxygenase-1 (IDO1) [81]. The immune response caused by food allergy abnormalities is the canine uridine pathway. A study reported that probiotic preparations consisting of *Bifidobacterium, Streptococcus thermophilus,* and *Lactobacillus* reduced plasma kynurenine (Kyn) concentrations and decreased IDO1 enzyme activity—in that way, regulating the kynurenine pathway [82].

### 3.6. Probiotics Improve Host Lipid Metabolism to Influence Immunity

A high-fat diet (HFD) causes obesity and increases food allergy incidence. Food allergic reactions induced by ovalbumin as an allergen in HFD mice revealed a surge in mast cell degranulation and the level of the helper T lymphocyte 2 (Th2) humoral response [83]. Reduced production of short-chain fatty acids (SCFAs) in the body is one of the distinctive features of obese patients [54,84]. SCFAs mainly contain acetate, propionate, and butyrate, of which acetate keeps the epithelial tissue of the infected intestinal organism relatively intact, thereby maintaining the health of the intestinal barrier and reducing the probability of food allergy [85]. Probiotics can produce acetate from pyruvate via the acetyl coenzyme A or anaerobic acetyl coenzyme A pathway, which reduces CO_2_ to CO to produce acetate [86]. At the same time, SCFAs affect Treg differentiation and B-cell production of sIgA and IL-22 by activating relevant signaling pathways of the immune system, reducing the intense immune response induced by food allergy.

Many anaerobic probiotics in the colon can use incompletely digested dietary fiber with resistant starch in the intestine and produce metabolites such as SCFAs [87]. *Lactobacillus paracasei* X11 and *Lactobacillus casei* YRL577 result in superior gastrointestinal tract viability by increasing SCFA metabolism [88]. A Korean study analyzed nutritional intake and allergenicity in children and reported that fat intake is usually associated with allergic diseases [89]. Long-chain fatty acids are lipid metabolites after digestion and absorption of food by the human body. Long-chain fatty acids can regulate the lipid metabolism of the cell membranes, accelerate tissue inflammation into the period of remission and repair, and thus achieve the effect of treating food allergy. Lipases produced by some probiotics can catalyze the synthesis of structural lipids of medium- and long-chain fatty acids [90]. Lipases produced by certain probiotic bacteria can catalyze the synthesis of structural lipids of medium and long-chain fatty acids [91]. Jointly, probiotics can improve the host’s resistance to food allergy by participating in host lipid metaboli0sm. They can use the incompletely metabolized substances of the host for secondary metabolism to produce SCFAs to regulate the immune response.

## 4. Postbiotics and Food Allergy

Probiotics can be effective in the treatment and prevention of food allergies, but there are limitations to these biologically active strains. In clinical treatment, we need to consider whether these probiotics can be transferred to the tissues and blood to trigger an inflammatory response. It is also important to consider whether the colonization pattern of probiotics may interfere with the normal colonization of the patient’s gut microbes [92]. Although this phenomenon is relatively rare, the safety risk caused by using probiotics in immunocompromised patients cannot be ignored. Second, some probiotics are unstable at room temperature and have a short shelf life for biological activity. In this regard, postbiotics are highly preferred, which have similar immune effects on living bacteria and are easy to store and transport [93].

Postbiotics are a type of microbial or component inhibitor beneficial to the host but lacking vitality. They have immunomodulatory effects and excel in anti-inflammatory, anti-tumour, and antioxidant actions [94]. The first type of postbiotic is dormant microbial cells that have been heat-treated or treated with radiation and high pressure [95]. The second is the cell-free supernatant derived by centrifuging the organism’s fermentation broth, which includes several fermentation products such as assemble-promoting factor, bacteriocins, and short-chain fatty acids. On the one hand, these components can modulate cytokine-mediated immune activity, safeguarding intestinal homeostasis and reducing excessive inflammation.

On the other hand, they can also enhance oral tolerance and prevent food allergy [96]. Experiments in mice have demonstrated that *Akkermansia muciniphila* helps to reduce TNF-α plasma levels and increase IL-10 in mice after pasteurization [97]. Clinical trials have demonstrated sublingual immunotherapy’s safety and clinical efficacy with heat-inactivated bacteria (MV130) for preventing respiratory tract infections [95]. However, in some cases, the postbiotic does not work as well as the live bacterium, such as when inactivated *Bifidobacterium longum* subsp. *longum* 5^1A^ immunity produced beneficial effects in inflammation in a mouse model of food allergy [98]. This suggests that live bacteria have advantages that are unmatched by postbiotics. It is undeniable that postbiotics possess a similar ability to intervene in food allergy as probiotics while minimizing the risk associated with ingestion. In the future, postbiotic interventions may be another relatively safe candidate for adjuvant therapy to prevent and treat food allergies.

## 5. Conclusions

Probiotics can play a vital role in the prevention and cure of all kinds of food allergy, and it has the potential to heal the immune system disorder caused by food allergy. Probiotics are closely associated with intestinal flora, which can colonize the host’s intestinal tract to regulate and keep stable intestinal flora. That way, it maintains the intestinal tract’s normal physiology, improves the intestinal cell wall barrier, and relieves food allergy symptoms. Probiotics participate in the metabolism of protein and lipids, improve host metabolism, and reduce the cycle of food allergy, which has a promising future in treating food allergy. However, the research scale of probiotics to treat food allergies is still miniature, and not all strains have preventive and therapeutic effects. Different strains produce different cytokines; many related safety and effectiveness issues must be considered. The probiotic’s potential for food allergy treatment is optimistic. The following in-depth research should be performed to realize the regulation and intervention of host immune regulation. The first goal is to understand the function of particular bacteria in the gut in food allergies and which kind of probiotics is more beneficial in preventing or treating food allergies. Second, expand the clinical study of probiotics on metabolic disorders to specifically investigate the effects of probiotics on the host brain–gut axis, choose dominant probiotics as the basic raw material of probiotics supplements, and then determine how these probiotics supplements improve health, whether it has the same effect on food allergies in all age groups, and whether different strains have different effects in treating food allergies. For postbiotics with a stronger safety profile, most of the current reports are about their potential to treat food allergies, and most studies do not consider the appropriate doses of different postbiotics to be administered. With the advancement of science and technology, we must also investigate how probiotics interact with the intestinal flora at the molecular level through the exchange of genetic material and further explore the prospects of probiotic applications in food allergy.

## Figures and Tables

**Table 1:** Allergenic proteins in food.

Food Source of Allergy	Sensitized Protein	Allergy Mechanism	Isoelectric Points	KD (ku)	Reference
Soybean	7S globulin	IgE, IgG co-mediate	8.70	190	[7]
Dairy products	Casein	IgG-mediated	4.60~6.00	14	[8]
	lactalbumin	IgE-mediated	4.80	14	[9]
Aquatic Products	Water-soluble Glycoprotein	IgE, IgG co-mediate	---	10~70	[9]
	Parvalbumin	IgE, IgG co-mediate	3.90~5.50	10~14	[10]
	Tropomyosin	IgE-mediated	4.50	38~40	[10]
	Aldolase A	IgE-mediated	---	40	[10]
	β-enolase	IgE-mediate	---	47~50	[11]
Egg products of poultry	Ovomucoid	IgE-mediated	3.83~5.50	28	[12]
	Ovalbumin	IgE-mediated	---	45	[13]
	Ovotransferrin	IgE-mediated	6.50	76.6	[13]
	Lysozyme	IgE-mediated	---	14.3	[13]
Nut products	Serum C—reactive protein	IgE-mediated	4.49~5.61	15~80	[14]

**Table 2:** Effect of probiotics on food-allergic hosts.

Probiotics	Experimental Model	Effect on Immunity	References
*Lactobacillus rhamnosus* CGMCC 1.3724	Peanut-allergic children	Decreased levels of peanut-specific IgE and increased levels of peanut-specific IgG	[47]
*Lactobacillus casei* CRL431 and *Bifidobacterium lactis* Bb-12	Children with milk allergies	The proportion of lymphocytes increased	[48]
*Lactobacillus casei* BL23	Cholera toxin-containing milk-sensitized mice	IL-17 secretion increased	[49]
*Lactobacillus reuteri*	Ovalbumin (OVA) sensitized BALB/c mice.	Allergic diarrhea, activation of mast cells, and the generation of serum IgE in allergic mice were alleviated.	[50]
*Lactobacillus plantarum* CJLP133	Intestinal allergy in 6-week-old BALB/c mice	It inhibited the production of IL-4, IL-5, IL-13, and IL-17A cytokines in the spleen cells of OVA-sensitized mice.	[51]
*Lactobacillus gasseri* OLL2809	OVA-sensitized mouse model	The proliferation rate and IL-2 production of CD4 T cells in OVA-fed mice were significantly reduced.	[52]
*Bifidobacterium infantis* 14.518	A mouse model sensitized to shellfish tropomyosin	Stimulated the maturation of DC cells and the accumulation of tolerant DC cells in intestinal-associated lymphoid tissues.	[53]
*Leuconostoc citreum* L3C1E7,	Ovalbumin-sensitized rat model.	Significantly reducing plasma OVA-specific lgE may alleviate Th2-mediated allergic symptoms.	[54]
*Clostridium butyricum* CGMCC0313-1	β-lactoglobulin (BLG) sensitized mouse model	SIgA increased, reversing the imbalance of Th1/Th2 and Th17/Treg.	[55]

## Data Availability

Research data are not shared.
